# HBV-DNA Load-Related Peritumoral Inflammation and ALBI Scores Predict HBV Associated Hepatocellular Carcinoma Prognosis after Curative Resection

**DOI:** 10.1155/2018/9289421

**Published:** 2018-09-20

**Authors:** Rui Liao, Cheng-You Du, Jian-Ping Gong, Fang Luo

**Affiliations:** ^1^Department of Hepatobiliary Surgery, The First Affiliated Hospital of Chongqing Medical University, Chongqing 400016, China; ^2^Chongqing Key Laboratory of Hepatobiliary Surgery, The Second Affiliated Hospital of Chongqing Medical University, Chongqing 400010, China; ^3^Department of Hepatobiliary Surgery, The Second Affiliated Hospital of Chongqing Medical University, Chongqing 400010, China

## Abstract

**Background:**

Both persistent inflammatory activity and liver function damage contribute to a poor prognosis of hepatocellular carcinoma (HCC). This study aimed to develop nomograms that incorporate hepatitis virus B (HBV)-related peritumoral inflammation score (PIS) and liver function based on ALBI score to predict postoperative outcomes of HCC.

**Methods:**

The prognostic roles of HBV-related preoperative PIS and ALBI scores in HCC recurrence were examined, and then two nomograms were constructed. The predictive accuracy and discriminative ability of the nomograms were compared with AJCC and BCLC staging systems of HCC.

**Results:**

PIS (HBV-PIS) and ALBI scores (HBV-ALBI) with different HBV-DNA loads had association with overall survival (OS) and/or recurrence-free survival (RFS) of HCC. The independent predictors of OS and RFS were incorporated into the corresponding nomograms. In the training cohort, the C-indexes of OS and RFS nomograms were 0.751 and 0.736, respectively. ROC analyses showed that both OS and RFS nomograms had larger AUC (0.775 and 0.739, respectively) than AJCC and BCLC staging systems. These results were verified by the internal and external validation cohorts.

**Conclusion:**

The proposed nomograms, including HBV-DNA load-related PIS and ALBI scores, were accurate in predicting survival for HCC after curative resection.

## 1. Introduction

Hepatocellular carcinoma (HCC) is a malignant tumor of the liver, primarily following infection by the hepatitis virus (HBV or HCV), and is ranked third among the top cancers by mortality worldwide. Currently, curative therapies (e.g., hepatectomy and liver transplantation) remain a first-line treatment option available to HCC patients, especially those with good liver functional reserve. Unfortunately, survival after a surgical resection is often jeopardized by the high rate of tumor recurrence (50-70% at 5 years) due to the lack of effective methods for early diagnosis and surveillance of anticancer treatment response [1]. Thus, it is reasonable to stratify patients at high risk of recurrence for appropriate treatment-related decisions, individualized monitoring, and follow-up after curative surgery.

HBV infection remains a major cause of HCC worldwide. A number of studies have demonstrated that HCC recurrence is related to HBV-related inflammatory activity, which promotes the proliferation of premalignant cells [2, 3]. High viral loads and Ishak hepatic inflammation score are associated with poor outcomes of HBV-related HCC after surgery [4, 5]. Of note, tumor progression is not exclusively the decisive element of the prognosis. Liver function, as well as alternative treatment, also significantly influences the survival of patients. Moreover, HBV-related inflammatory activity may regulate both the status of liver dysfunction and the tumor biology in patients with HCC. Therefore, large-scale studies with comprehensive clinical data that illustrate HBV-associated inflammation and hepatic function scores in HCC patients are substantial.

ALBI score is a newly emerging alternative to the conventional Child-Pugh (C-P) score for grading liver function because it is evidence-based and easier to implement, involving only albumin and bilirubin [6]. When integrated into the Barcelona Clinic Liver Cancer (BCLC) system or Japan Integrated Staging (JIS), ALBI showed similar or even better prognostic performance than the C-P score [7]. ALBI is proposed to be both an objective measurement of hepatic function reserve and a predictor of HCC prognosis after surgery.

Unfortunately, the risk of peritumoral inflammation and the relationship of ALBI scores with viral replication status after curative hepatectomy for HBV-related HCC are still underreported. Currently, investigations about the relationship between tumor recurrence and these risk factors are absent.

In this study, we specifically looked for the association between HBV-related inflammatory status and the prognosis of patients with HCC after surgery. We developed two reliable nomograms comprising peritumoral inflammation (PIS) and ALBI scores with different HBV-DNA loads for patients who underwent curative resection. These nomograms could provide us a more accurate estimation of outcomes in routine clinical practice and a better understanding of the long-term HBV-related inflammatory impact on the survival of patients with HCC.

## 2. Materials and Methods

### 2.1. Patient Selection and Follow-Up Procedure

We accrued retrospective data from the First Affiliated Hospital of Chongqing Medical University between January 2009 and December 2011. A total of 512 consecutive HCC patients undergoing curative resection were selected for retrospective analysis. Of these selected, 46 patients were excluded, according to the inclusion and exclusion criteria described previously [8]. Briefly, (1) all patients tested HBV surface antigen (HBsAg) and HBV-DNA loads; (2) reliable laboratory test data including liver function; (3) the absence of preoperative extrahepatic metastases confirmed by computed tomography (CT) and/or magnetic resonance imaging (MRI) scanning; (4) no preoperative anticancer therapies; (5) complete resection of all tumor nodules; (6) complete patient records and follow-up data; (7) survival for more than 30 days after surgery. Patients were excluded if they had any infection and autoimmune disease or anticancer therapies before operation. Finally, 466 patients qualified for this study and were divided into a training cohort (n=342, from January 2009 to June 2011) or an internal validation cohort (n=124, from July 2011 to December 2011, Table 1). To serve as an external validation cohort, another independent cohort of 186 consecutive patients with histologically proven HCC after surgery selected from the Second Affiliated Hospital of Chongqing Medical University between January 2008 and July 2009. This study was approved by the Ethics Review Committee of the First and Second Affiliated Hospital of Chongqing Medical University. Informed consent was obtained from all patients before surgery.

There were 538 patients with positive HBsAg. 98 HBV-HCC patients received regular antiviral therapy after surgery for more than 90 days. The starting time was within 6 months after surgery. They began receiving oral nucleoside/nucleotide analogs therapy within 1 week after surgery until HBsAg seroconversion. Adefovir (10 mg/day), entecavir (0.5 mg/day), or lamivudine (100 mg/day) was recommended. All patients had the following follow-up schedule: once a month for the first 6 months postsurgery, then every 2-3 months until a year postsurgery, and finally, once every 6 months. A routine examination was conducted for each follow-up, including serum alpha-fetoprotein (AFP), serum biochemistry, abdomen ultrasonography, chest X-ray, or abdominal CT or MRI examination. A detailed clinical history and physical examination were recorded. Overall survival (OS) was defined as the interval between the date of the first surgery and the date of death or the last follow-up for surviving patients. Recurrence-free survival (RFS) was defined as the interval between the date of surgery and the date of recurrence for relapsed patients or the last follow-up for nonrecurrent patients.

### 2.2. Tissue Microarray and Evaluation of Necroinflammatory Activity

Hematoxylin and eosin (H&E) was performed in a tissue microarray (TMA), as described previously [9, 10]. Triplicate cores of 1 mm were taken from each formalin-fixed, paraffin-embedded surgical specimen of the corresponding nonnecrotic tissue. Peritumoral tissue was 1.5 cm from the border of HCC tissues. The assessment of PIS was based on the extent and distribution of the predominantly inflammatory infiltrate characteristic as described by Ishak et al., including portal, periportal, and intra-acinar inflammatory cell infiltration, and liver cell necrosis. Briefly, necroinflammatory activity in the liver tissues was divided into four levels: Grade 1 (1-4), no activity; Grade 2 (5–8), mild; Grade 3 (9–12), moderate; and Grade 4 (13–18), severe [11].

### 2.3. Calculation of the ALBI Score

The ALBI score was defined as follows: 0.66 × log_10_ (total bilirubin *μ*mol/L) – 0.085 x (albumin g/L). Patients were stratified into three groups according to specific cutoffs of the original publication: Grade 1, ≤-2.60; Grade 2, >-2.60 to -1.39; and Grade 3, >-1.39 [6]. All blood samples were obtained two days before surgery.

### 2.4. Statistical Analysis

Categorical variables were compared using the* χ*^2^ test or Fisher's exact test. Continuous variables were compared using Student's t-test or the nonparametric Mann–Whitney* U* test. The correlation between variables was analyzed with Pearson's or Spearman's *ρ* coefficients test. Receiver operating characteristics (ROC) curves were defined by sensitivity and specificity. The survival curves were determined by the Kaplan–Meier analysis and compared by the log-rank test. The Cox proportional hazards regression model was used to perform univariate and multivariate analyses in the training cohort.

Final model selection for nomograms was built by a backward step-down selection process based on the results of multivariable analyses of OS/RFS in the training cohort by using the “rms” package in R software, version 3.4.0 (http://www.r-project.org/) [12]. The discrimination of the nomograms was evaluated by concordance index (C-index) and assessed by calibration, which compared predicted survival by the Kaplan–Meier curves of the quartiles of predictions. The values of the C-index ranged from 0.5 (no discrimination) to 1.0 (perfect discrimination) [13]. Bootstraps with 1000 resample were used for both the validation of nomograms and the calibration assessment. Statistical analyses were performed with SPSS 19.0 (SPSS Inc., Chicago, IL, USA). All statistical tests were two-tailed, and a p value <0.05 was considered statistically significant. ROC curve analysis was used to compare OS/RFS prediction of the prognostic nomograms with those of the American Joint Commission on Cancer (AJCC) seventh edition [14] and Barcelona Clinic Liver Cancer (BCLC) [15].

## 3. Results

### 3.1. Baseline Characteristics

The baseline characteristics of the 652 patients included in this study are described in Table 1. The training cohort comprised 293 men and 49 women (median age, 52.5). The internal validation and external validation cohorts comprised 105 and 159 men and 19 and 27 women (median age, 51.5 and 53.0, respectively), respectively. In the three cohorts, the majority of patients had cirrhosis (training: 88.0%; internal and external validation: 82.3% and 88.2%, respectively) and slightly higher medium values of gamma-glutamyl transpeptidase (GGT; training: 55 U/L; internal and external validation: 57 and 56.5 U/L, respectively) than normal (50 U/L). Most patients had a single tumor (training: 88.0%; internal and external validations: 90.3% and 88.2%, respectively) of small size (≤3.0 cm; training: 36.0%; internal and external validation: 66.9% and 36.0%, respectively). Microvascular invasion occurred in approximately one-fourth of patients in both cohorts (training: 25.7%; internal and external validation: 28.2% and 27.4%, respectively). According to the TNM staging system, 21.6% (74/342) of patients in the training cohort and 26.6% (33/124) and 30.1 (56/186) of patients in the internal and external validation cohorts had a stage IIIA tumor. The baseline clinic pathological characteristics were broadly similar among the three cohorts (most P>0.05).

**Figure 1:**
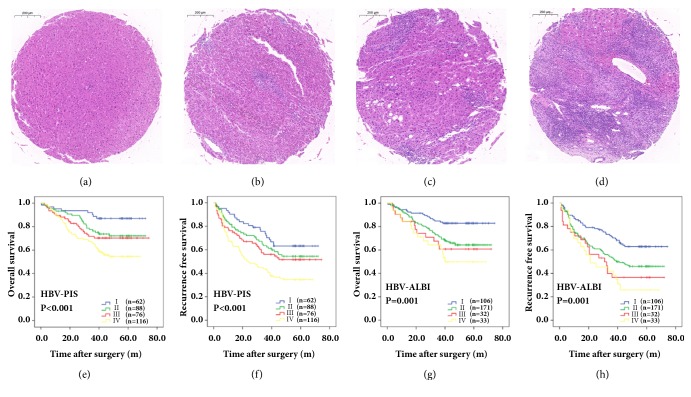
Four levels of inflammatory infiltrate according to Ishak score (a-d). HBV-DNA load related peritumoral inflammatory score (HBV-PIS) and ALBI score (HBV-ALBI) are associated with overall survival (OS) and recurrence-free survival (RFS) of patients with hepatocellular carcinoma (HCC) in training cohort, respectively (e-h).

We detected PIS according to Ishak score and found four levels of inflammatory infiltrate, including 34, 23, and 91 patients with Grade 1, 119, 36, and 24 patients with Grade 2, 141, 26, and 51 patients with Grade 3, and 48, 39, and 20 patients with Grade 4 in the training cohort and the internal and external validation cohorts, respectively (Figures 1(a)–1(d)).

In this present study, no patients with an ALBI score >-1.39 were observed. For the training and internal and external validation cohorts, 81.0% (277/342), 56.5% (70/124), and 78.0% (145/186) of patients had an ALBI score ≤-2.60, respectively.

### 3.2. Association of HBV-Related PIS and ALBI Scores with OS and RFS in the Three Cohorts

In the total of 652 patients, both PIS and ALBI were associated with the outcomes of HCC after surgery (Figure S1). To study the influence of viral replication status on the PIS and ALBI scores, as well as their association with the outcomes of HCC after surgery, patients were divided into four subgroups based on PIS and ALBI score according to HBV-DNA loads: I: low PIS (Grade 1-2)/ALBI (Grade 1) with low HBV-DNA loads; II: low PIS/ALBI with high HBV-DNA loads; III: high PIS (Grade 3-4)/ALBI (Grade 2) with low HBV-DNA loads and IV: high PIS/ALBI with high HBV-DNA loads. Group IV (high PIS or high ALBI with high HBV-DNA loads) was related to poor OS and RFS in the training cohort (P<0.001 and =0.001 for OS, P<0.001 and =0.001 for RFS, respectively; Figures 1(e)–1(h)) and the internal (P=0.04 and P<0.001 for OS, both P<0.001 for RFS, respectively) and external validation cohorts (P<0.001 and =0.001 for OS, P<0.001 and =0.001 for RFS, respectively; Figure S2). In the total of 652 patients, HBV-ALBI (OS: 0.689, RFS: 0.642) or HBV-PIS (OS: 0.746, RFS: 0.603) is better in predicting the prognosis of HCC than ALBI (OS: 0.526, RFS: 0.586) or PIS (OS: 0.562, RFS: 0.588, Figure S3).

### 3.3. Independent Prognostic Factors and Development of OS and RFS Nomograms in the Training Cohort

**Figure 2:**
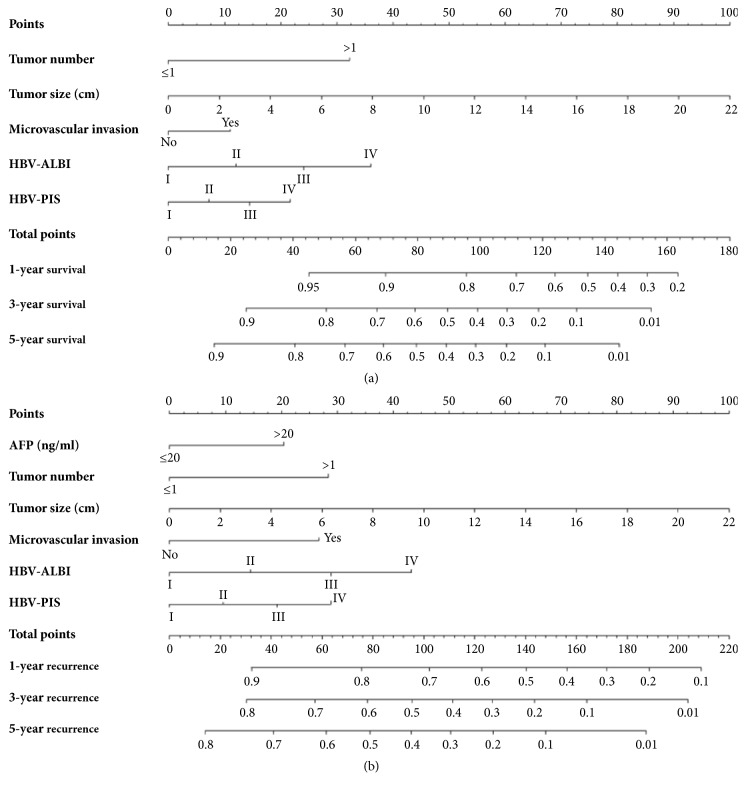
Nomograms for predicting survival of hepatocellular carcinoma patients after hepatectomy. To calculate the probability of overall survival (OS, (a)) and recurrence-free survival (RFS, (b)), straight upward lines are drawn to determine the points accrued. The sum of these points is plotted on the total points bar to the probability to yield the 1-, 3-, and 5-year survival or recurrence rates.

In the training cohort, the mean OS was 42.3 months (range, 1.0-74.3 months), and the 1-, 3-, and 5-year OS rates were 81.5%, 65.6%, and 45.2%, respectively. The mean RFS was 35.9 months (range, 1.0-74.3 months). The 1-, 3-, and 5-year RFS rates were 65.4%, 49.0%, and 31.3%, respectively. After univariate analyses of our data, multivariate analyses were performed on significant clinical factors. These analyses demonstrated that tumor number (P=0.004), tumor size (P<0.001), microvascular invasion (P=0.046), HBV-PIS (P=0.004), and HBV-ALBI (P=0.007) were independent prognostic factors of OS. In addition, AFP (P=0.024), tumor number (P=0.027), tumor size (P<0.001), microvascular invasion (P=0.007), HBV-PIS (P=0.042), and HBV-ALBI (P=0.006) were independent prognostic factors of RFS (Table 2). Furthermore, the independent risk factors of OS (Figure 2(a)) and RFS (Figure 2(b)) were incorporated into the nomograms.

### 3.4. Predictive Performance of the Nomograms in the Training Cohort

**Figure 3:**
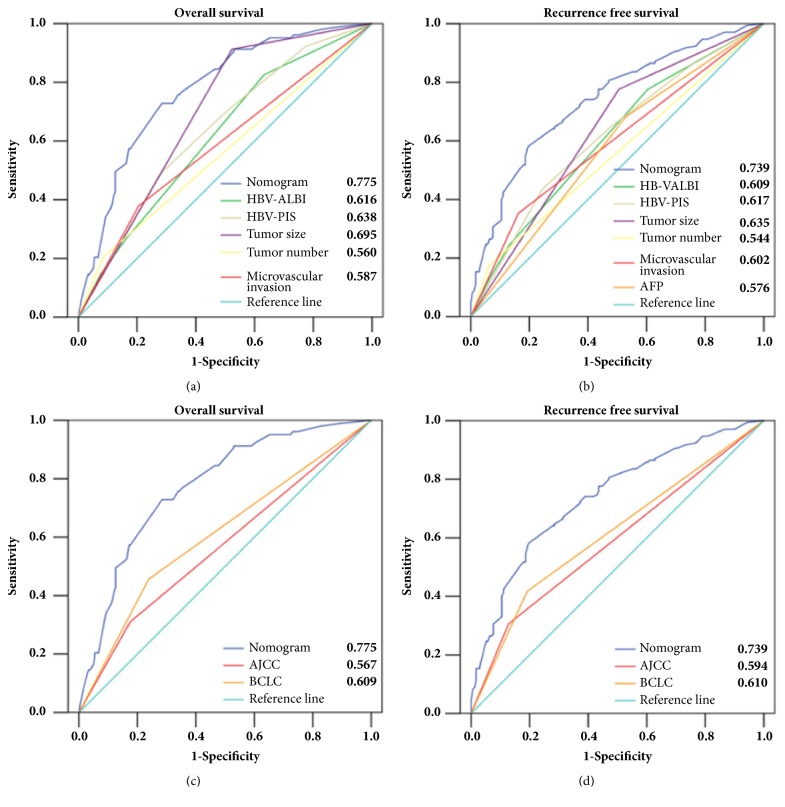
Predictive accuracy comparison of each variable included in the nomograms ((a) and (b)) and comparison between the nomograms and two conventional clinical staging systems (AJCC and BCLC staging systems, (c) and (d)) by ROC curve analyses for overall survival (OS, (a) and (c)) and recurrence-free survival (RFS, (b) and (d)) in the training cohort, respectively. The numbers shown in right lower part of each panel (a-d) represent the area under receiver operating characteristics curves of the parameters.

**Figure 4:**
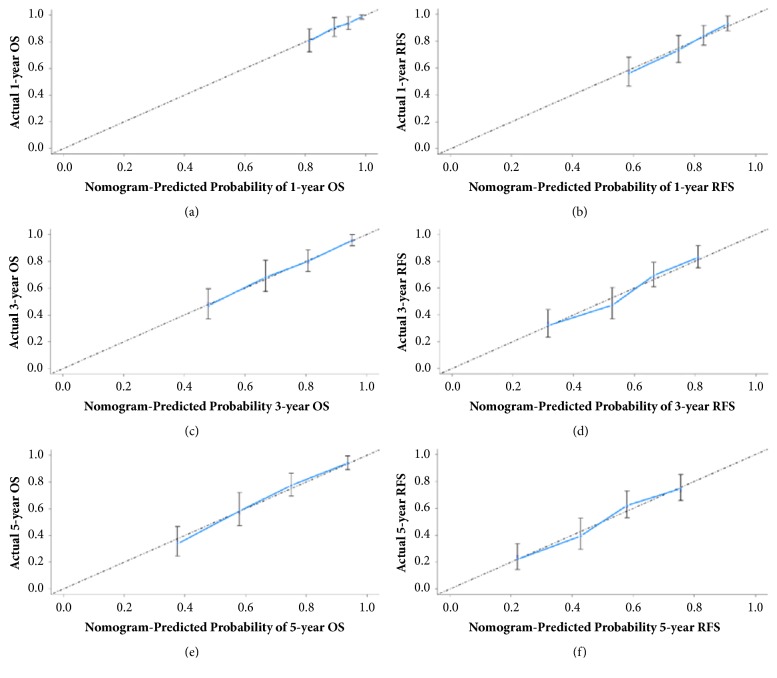
The calibration curves for predicting 1- ((a) and (b)), 3- ((c), and (d)) and 5-year ((e) and (f)) overall survival (OS, (a), (c) and (e)) and recurrence-free survival (RFS, (b), (d), and (f)) rates by nomograms prediction and actual observation in patients with hepatocellular carcinoma in the training cohort, respectively.

The C-indexes of the OS and RFS nomograms were 0.751 (95% CI: 0.707-0.795) and 0.736 (95% CI: 0.701-0.770), respectively, which were higher than any other independent risk factor incorporated into the nomograms (Table 3). Similarly, by ROC analyses, OS and RFS nomograms also showed the largest AUC (0.775 and 0.739) compared to other risk factors included in the nomograms (Figure 3). The calibration plot for the probability of 1-, 3-, or 5-year OS and RFS after surgery had an optimal agreement between the nomograms for probabilities and the actual observations in the training cohort (Figure 4).

Meanwhile, both AJCC and BCLC staging systems predicted the survival of HCC after surgery (Figure S4). As shown in Table 3, the C-indexes of OS and RFS nomograms were significantly higher than AJCC seventh edition stage (0.543 and 0.537) and BCLC stage (0.603 and 0.591). In addition, by ROC analyses, OS and RFS nomograms showed the largest AUC compared to these two conventional clinical staging systems (all P<0.001, Figure 3). The results suggest that the two nomograms were accurate predictors for OS and RFS in patients with HCC after curative resection.

### 3.5. Validation for the Nomograms

In the internal and external validation cohorts, the mean OS was 39.3 months (range, 1.0-60.0 months) and 40.1 months (range, 1.5-82.2 months), respectively, and the 1-, 3-, and 5-year OS rates were 80.2% and 81.8%, 60.5% and 62.5%, and 43.1% and 44.5%, respectively. The mean RFS was 33.9 months (range, 1.0-60.0 months) and 33.7 months (range, 1.0-79.3 months), respectively. The 1-, 3-, and 5-year RFS rates were 62.3% and 61.5%, 43.1% and 42.5%, and 28.4% and 38.2%, respectively. The C-index of the nomograms for predicting OS and RFS was 0.817 (95% CI: 0.860-0.796) and 0.799 (95% CI: 0.737-0.862), and 0.802 (95% CI: 0.835-0.7769) and 0.704 (95% CI: 0.654-0.754), respectively. ROC analyses showed OS and RFS nomograms had a larger AUC than any other independent risk factor or the two clinical staging systems mentioned above (all P<0.001, Figure S5). Calibration curves of the nomograms showed good agreement between prediction and observation in the probability of 1- and 3- or 5-year recurrence (Figures S6 and S7). In the total of 652 patients, ROC analyses also showed OS and RFS nomograms had a large AUC (OS: 0.831 and RFS: 0.806, respectively, Figure S8).

### 3.6. Antiviral Treatment

After antiviral treatment, no serious adverse event was observed. In the 98 HBV treated patients, 92 (93.9%) patients had persistent undetectable HBV-DNA (>10^3^ copies/ml) until the last follow-up. There were 4 patients who had a primary nonresponse to antiviral drug at month 3 or 6. Only 1 patient had HBsAg seroconversion.

To investigate the association between nucleoside/nucleotide analogs therapy and prognosis of patients with HBV-related HCC after curative surgery, 538 patients were divided into two subgroups: treated cohort (n=98) and untreated cohort (n=440). The majority of baseline clinicopathological characteristics had no significant difference between the two cohorts (Table 4). Compared with the untreated cohort, the treated cohort had better OS (44.8±15.7 versus 38.5±19.4 months, P=0.003) and RFS (37.4±21.9 versus 32.2±21.6 months, P=0.034). Among 538 HBV-related HCC patients, univariate analysis did not show that nucleoside/nucleotide analogs therapy could predict OS (P=0.233) and RFS (P=0.186) of HCC patients.

## 4. Discussion

Persistent inflammation activity is considered to be one of the important risk factors for the tumor prognosis of HCC. Of note, the early recurrence of HCC is possibly related to the dissemination of primary HCC tumor cells induced by the inflammatory response [16]. In this study, we demonstrated that high PIS and ALBI scores with high HBV-DNA loads had the prognostic capacity for the poor outcomes of HCC after surgery. Moreover, we constructed two novel nomograms comprising HBV-PIS, HBV-ALBI, AFP, tumor number, tumor size, and microvascular invasion, which allowed accurate prognostic prediction for OS and RFS of a patient with HCC after curative resection.

In the present study, HBV-PIS and HBV-ALBI were utilized because both values are objective serology-based predictive models for HCC survival. Previous studies have reported that a high Ishak inflammation score is an associated risk factor for postoperative HBV reactivation [5]. In China, the majority of patients with HCC were infected by HBV and developed HCC from fibrosis and cirrhosis, suggesting a highly etiological connection among these diseases. Moreover, high HBV viral loads may affect the prognosis of HBV-related HCC patients. On the one hand, active viral replication in hepatocytes is associated with more liver dysfunction, which subsequently limits the therapeutic options for HBV-related HCC. On the other hand, the active inflammation induced by HBV in the liver parenchyma increases the intrahepatic metastasis and/or de novo tumor development by mediating alteration in some molecules and factors to carcinogenesis and thereby changing the tumor microenvironment [17, 18]. Furthermore, high HBV loads may be a driving force of active necroinflammation and HBV mutants, which promote the invasive ability and metastatic potential of HCC [19, 20].

After being combined with HBV-DNA loads, both PIS and ALBI showed better predictive powers for OS and RFS of HCC patients after curative resection, revealing the ongoing impact of HBV on the liver tissues (such as liver local inflammation/immune response and hepatic function damage, et al) and subsequently becoming a major contributor in hepatocarcinogenesis to affect the prognosis of tumor hosts [21, 22]. We found that low HBV-DNA loads significantly predict good prognosis in univariate analysis but became nonsignificant in multivariate analysis. One of the reasons may be associated with antiviral therapy which made some patients with high HBV-DNA loads into low HBV-DNA loads and thereafter got better prognosis. These changes affected the predictive power of HBV-DNA loads. HBV-DNA loads, HBV-ALBI, HBV-PIS, and BCLC staging were statistically different between the training and validation cohorts, but the two nomograms still could accurately predict the outcomes of HCC after surgery, possibly suggesting the wild application of the models in various surgical HCCs with different HBV infection status and stage.

However, HBV-PIS and HBV-ALBI are not perfect predictors with high sensitivity and specificity for predicting outcomes of HCC according to ROC analysis. Therefore, we developed the two nomograms that could provide accurate prognosis information (AUC: 0.775 for OS and 0.739 for RFS). Of all risk factors incorporated into the nomograms, preoperative AFP level, tumor number, tumor size, and microvascular invasion have been demonstrated to be associated with surgical prognosis of HCC [23–26]. Our multivariate analysis revealed that HBV-PIS and HBV-ALBI also had prognostic value for this prognosis. These findings are likely due to the extremely heterogeneous inflammation microenvironment of HCC, which is influenced by tumor characteristics, liver function, and hepatitis activity. Our previous study suggested the peritumoral liver tissue is indisputably the principal target organ for the recurrence of HCC [27, 28] and related to the aggressiveness of the tumor [3, 29]. Therefore, including HBV-PIS and HBV-ALBI into the two nomograms may contribute to a significantly increased predictive accuracy due to the close relationship between tumor development and inflammatory response underlying liver diseases. Although AJCC and BCLC staging systems showed the abilities to stratify patients after surgery into distinct risk categories, the two nomograms performed better predictive accuracy for outcomes of HCC. In the training and validation cohorts, the C-index, the calibration curve, and ROC analysis supported that our nomograms were superior to the two conventional staging systems. Here, AFP has not been found as a predictor of overall survival (OS). On the one hand, many clinical studies demonstrated that low AFP levels of ≤400 ng/ml were a significant favorable prognostic factor for HCC. Nevertheless, 30%-40% of HCC patients with low serum AFP concentration (≤400 ng/ml) were difficult to monitor. Here, we used 20ng/ml as the cutoff value of AFP which may decrease their predictive value in OS. On the other hand, considering the relative small ratio of sample size (59.4% and 30.7% for the patients with AFP >20 and >400 ng/ml, respectively), a conclusion about the predictive role of high AFP levels in HCC should be cautiously interpreted.

So far, studies regarding the effectiveness of antiviral therapy in HCC prognosis have produced conflicting results [30, 31]. In this study, our data showed that the treated cohort had better OS and RFS compared with the untreated cohort. However, antiviral therapy was not an independent predictive factor of outcomes of HCC patients. Here, considering such a small sample size receiving antiviral therapy (n=98), a conclusion about the effectiveness of antiviral therapy in HCC prognosis should be cautiously interpreted. Moreover, a part of patients with late and short-term antiviral therapy were not in time and had inadequate duration to control the activation of viral hepatitis B and would probably facilitate HCC recurrence [31]. Therefore, the majority of patients with HBV in this study did not achieve effective antiviral response. Furthermore, 99.7% surgical patients had good liver function (C-P Grade A) to meet liver resection requirements and there is no difference in liver function between antiviral treated and untreated patients (Table S2). Irreversible cirrhosis induced by preoperative HBV is still associated with histologic necroinflammation after surgery although viral replication inhibition could partially improve the necroinflammatory activity. Before liver resection, some environmental exposure including viral infection and gene products involved in an inflammatory response could lead to an increased potential for future malignant transformation [32]. Thus, we thought it was reasonable in this study that HBV-PIS and HBV-ALBI had prognostic value for HCC prognosis after curative resection.

There are several limitations in the present study. A regular assessment of postoperative HBV viral reactivation during the follow-up was not performed due to the incomplete data. In addition, further studies are necessary to validate the predictive nomogram in the patients without surgery after neoadjuvant or adjuvant therapies. The nomograms may not be suitable for HCC patients with etiologies outside of HBV infection, especially for a Western population who are mainly infected by HCV.

## 5. Conclusions

In summary, we have developed two objective reliable nomograms to predict postoperative survival of patients with HCC. These tools may help us make informed decisions for the early diagnosis of HCC and prevention of recurrence following curative resection. A large-scale prospective validation study is needed to determine whether these tools can be applied widely.

## Figures and Tables

**Table 1:** Clinical backgrounds of all patients and comparisons between the training and validation cohorts.

**Characteristics**	**Total**	**Training cohort**	**Internal validation cohort**	**External validation cohort**	**P-value** **∗**
	**n=652**	**n=342**	**n=124**	**n=186**	
Age, yr, median, (range)	52.5 (18.0-77.0)	52.5 (18.0-75.0)	51.5 (21.0-77.0)	53.0 (18.0-75.0)	0.293
Gender (Female/Male)	95/557 (14.6%/85.4%)	49/293 (14.3%/85.7%)	19/105 (15.3%/84.7%)	27/159 (14.5%/85.5%)	0.964
Cirrhosis (yes/no)	567/85 (87.0%/13%)	301/41(88.0%/12.0%)	102/22(82.3%/17.7%)	164/22 (88.2%/11.8%)	0.249
ALT U/L, median (range)	40.0 (8.0-912.0)	39.0 (9.0-912.0)	37.5 (10.0-297.0)	41.5 (8.0-835.0)	0.369
AST, U/L, median (range)	36.5 (7.0-527.0)	36.0 (7.0-523.0)	40.0 (12.0-205.0)	36.0 (11.0-527.0)	0.587
GGT, U/L, median (range)	56.5 (7.0-624.0)	55.0 (8.0-583.0)	57.0 (9.0-624.0)	56.5 (7.0-388.0)	0.163
ALB, g/L, median (range)	43.0 (28.0-59.0)	43.0 (30.0-57.0)	42.5 (28.0-59.0)	43.0 (33.0-55.0)	0.276
TBIL, *μ*mol/L, median (range)	13.8 (2.6-135.5)	13.7 (4.6-135.5)	13.9 (2.6-48.9)	13.1 (4.7-95.3)	0.258
Child-Pugh grade (A/B)	650/2 (99.7%/0.3%))	340/2 (99.4%/0.6%)	124/0 (100%/0)	186/0 (100%/0)	0.403
HBV-DNA loads	207/445 (41.7%/58.3%)	138/204 (40.4%/59.6%)	26/98 (21.0%/79.0%)	43/143 (23.1%/76.9%)	**<0.001**
(≤10^4^/>10^4^ copies/ml)					
Antiviral therapy (yes/no)	98/554 (15.0%/85%)	53/289 (15.5%/84.5%)	20/104 16.1%/83.9%)	25/161 (13.4%/86.6%)	0.218
HBV-ALBI (I/II/III/IV)	266/332/53/101	106/171/32/33 (31.0%/50.0%/9.4%/9.6%)	14/56/13/41	46/105/8/27	**<0.001**
	(40.8%/50.9%/8.1%/15.5%)		(11.3%/45.2%/10.5%/33.0%)	(24.7%/56.5%/4.3%/14.5%)	
HBV-PIS (I/II/III/IV)	105/219/102/226	62/88/76/116 (18.1%/25.7%/22.2%/34.0%)	16/43/11/54	27/88/15/56	**<0.001**
	(16.1%/33.6%/15.6%/34.7%)		(12.9%/34.7%8.9%/43.5%%)	(14.5%/47.3%/8.1%/30.1%)	
AFP, ng/ml, (≤20/>20)	265/387 (40.6%/59.4%)	136/206(39.8%/60.1%)	50/74(40.3%/59.7%)	79/107 (42.5%/57.5%)	0.798
Platelet, 10^9^/L, median (range)	138.0 (10.0-417.0)	137.0 (25.0-417.0)	137.5 (10.0-331.0)	145.0 (46.0-333.0)	0.139
HBsAg (Positive/Negative)	538/114 (82.5%/17.5%))	285/57 (83.3%/16.7%)	100/24 (80.6%/19.4%)	153/33 (82.3%/17.7%)	0.792
Tumor number (single/multiple)	578/74 (88.7%/11.3%)	301/41 (88.0%/12.0%)	113/11 (91.1%/8.9%)	164/22 (88.2%/11.8%)	0.626
Microvascular invasion (yes/no)	184/468 (28.2%/71.8%))	88/254 (25.7%/74.3%)	35/89 (28.2%/71.8%)	51/135 (27.4%/72.6%)	0.152
Tumor capsule(complete/Inomplete)	358/294 (54.9%/45.1%)	188/154 (55.0%/45.0%)	70/54 (56.5%/43.5%)	100/86 (53.8%/46.2%)	0.089
Tumor size, cm (≤3/>3)	225/427 (34.5%/65.5%)	123/219 (36.0%/64.0%)	35/89 (28.2%/71.8%)	67/119 (36.0%/64.0%)	0.123
AJCC stage (I-II/IIIA)	489/163 (75.0%/25.0%)	268/74 (78.4%/21.6%)	91/33 (73.4%/26.6%)	130/56 (69.9%/30.1%)	0.085
BCLC stage (0-A/B-C)	418/234 (66.3%/33.7%)	238/104 (69.6%/30.4%)	83/41 (66.9%/33.1%)	97/89 (52.2%/47.8%)	**<0.001**

ALT: alanine aminotransferase; AST: aspartate aminotransferase; GGT: gamma-glutamyl transpeptidase; ALB: Albumin; TBIL: total bilirubin; HBV-ALBI: hepatitis B virus load related ALBI score; HBV-PIS: hepatitis B virus load related peritumoral inflammatory score; AFP: alpha fetoprotein; HBsAg: hepatitis B virus surface antigen; AJCC: American Joint Committee on Cancer; BCLC: Barcelona Clinic Liver Cancer.

**Table 2:** Independent risk factors predicting prognosis of HCC in training cohort.

**Variables**	**Univariate analysis**		**Multivariate analysis**	
	**HR (95**%**CI)**	**P-value**	**HR (95**%**CI)**	**P-value**
**OS**				
Age	-	0.206		NA
Gender	-	0.836		NA
Cirrhosis	-	0.075		NA
ALT	-	0.509		NA
AST	-	0.057		NA
TBIL	-	0.362		NA
Platelet	-	0.283		NA
HBsAg	-	0.623		NA
Child-Pugh grade	-	0.386		NA
Antiviral therapy		0.863		NA
Tumor capsule	-	0.076		NA
GGT (U/L)	1.678 (1.132-2.486)	**0.009**	_	0.195
ALB (g/L)	0.640 (0.432-0.950)	**0.026**	_	0.443
AFP (≤20/>20 ng/ml)	1.581 (1.041-2.399)	**0.030**	_	0.190
Tumor number (single/multiple)	2.299 (1.422-3.717)	**<0.001**	2.051 (1.266-3.321)	**0.004**
Microvascular invasion (yes/no)	2.132 (1.431-3.177)	**<0.001**	1.431 (1.042-2.015)	**0.046**
Tumor size (≤3.0/>3.0 cm)	2.881 (1.751-4.743)	**<0.001**	3.125 (2.079-3.884)	**<0.001**
HBV-ALBI (I/II/III/IV)	2.499 (1.109-3.342)	**0.001**	2.539 (1.056-3.217)	**0.007**
HBV-PIS (I/II/III/IV)	2.512 (1.335-3.718)	**0.001**	2.859 (1.207-3.623)	**0.004**
**RFS**				
Age	-	0.053		NA
Gender	-	0.485		NA
Cirrhosis	-	0.063		NA
ALB	-	0.180		NA
ALT	-	0.647		NA
TBIL	-	0.713		NA
Platelet	-	0.478		NA
HBsAg	-	0.073		NA
Child-Pugh grade	-	0.825		NA
Antiviral therapy		**0.016**		0.112
Tumor capsule	-	0.170		NA
AST (U/L)	1.427 (1.048-1.943)	**0.023**	_	0.237
GGT (U/L)	1.464 (1.074-1.996)	**0.015**	_	0.089
AFP (≤20/>20 ng/ml)	1.679 (1.204-2.340)	**0.002**	1.458 (1.051-2.021)	**0.024**
Tumor number (single/multiple)	1.792 (1.183-2.713)	**0.005**	1.578 (1.054-2.363)	**0.027**
Microvascular invasion (yes/no)	2.197 (1.593-3.029)	**<0.001**	1.581 (1.136-2.199)	**0.007**
Tumor size (≤3.0/>3.0 cm)	1.548 (1.775-3.658)	**<0.001**	2.039 (1.404-2.962)	**<0.001**
HBV-ALBI (I/II/III/IV)	2.382 (1.582-2.816)	**0.001**	1.959 (1.112-2.785)	**0.006**
HBV-PIS (I/II/III/IV)	2.341 (1.464-2.544)	**<0.001**	2.008 (1.305-2.957)	**0.042**

HCC: hepatocellular carcinoma; HR: hazard ratio; CI: confidence interval; ALT: alanine aminotransferase; AST: aspartate aminotransferase; TBIL: total bilirubin; HBsAg: hepatitis B virus surface antigen; GGT: gamma-glutamyl transpeptidase; ALB: Albumin; AFP: alpha-fetoprotein; HBV-PIS: hepatitis B virus load related peritumoral inflammatory score; HBV-ALBI: hepatitis B virus load related ALBI score. NA: no adopted

**Table 3:** The C-index of the predictors in OS and RFS nomograms and clinical staging systems.

Variables	**C-index**	**95**%** CI**
OS		
Nomogram	0.751	0.707-0.795
Tumor size	0.662	0.629-0.696
HBV-PIS	0.621	0.572-0.634
HBV-ALBI	0.616	0.566-0.666
BCLC stage	0.603	0.561-0.650
Microvascular invasion	0.585	0.539-0.631
Tumor number	0.553	0.517-0.589
AJCC stage	0.543	0.504-0.582
**RFS**		
Nomogram	0.736	0.701-0.770
Tumor size	0.617	0.650- -0.584
HBV-PIS	0.596	0.549-0.643
HBV-ALBI	0.591	0.551-0.631
BCLC stage	0.591	0.554-0.628
Microvascular invasion	0.583	0.548-0.618
AFP	0.562	0.525-0.599
AJCC stage	0.537	0.566-0.508
Tumor number	0.531	0.506-0.556

OS: overall survival; RFS: recurrence free survival; AFP: alpha fetoprotein; HBV-PIS: hepatitis B virus load related peritumoral inflammatory score; HBV-ALBI: hepatitis B virus load related ALBI score; AJCC: American Joint Committee on Cancer; BCLC: Barcelona Clinic Liver Cancer.

**Table 4:** Clinical backgrounds of HBV-related hepatocellular carcinoma (n=538).

**Characteristics**	**Treated cohort (n=98)**	**Untreated cohort (n=440)**	**P-value**
Age, yr, median, (range)	52.2 (22.0-75.0)	52.1 (19.0-75.0)	0.747
Gender (Female/Male)	12/86 (12.2%/87.8%)	64/376 (14.5%/85.5%)	0.633
Cirrhosis (yes/no)	83/15(84.7%/15.3%)	385/55 (87.5%/12.5%)	0.506
ALTU/L, median (range)	42.0 (9.0-561.0)	39.0 (8.0-848.0)	0.410
AST, U/L, median (range)	37.0 (15.0-517.0)	36.0 (10.0-527.0)	0.957
GGT, U/L, median (range)	55.0 (16.0-610.0)	59.0 (7.0-624.0)	0.970
ALB, g/L, median (range)	43.0 (28.0-59.0)	43.0 (34.0-52.0)	0.276
TBIL, *μ*mol/L, median (range)	13.6 (6.2-45.3)	13.2 (2.6-130.0)	0.176
Child-Pugh grade (A/B)	97/1 (99.0%/1.0%)	439/1 (99.8%/0.2%)	0.331
HBV-DNA load	35/63 (35.7%/64.3%)	147/293 (33.4%/66.6%)	0.723
(≤10^4^/>10^4^ copies/ml)			
HBV-ALBI (I/II/III/IV)	29/54/6/9	118/211/40/71	0.213
	(29.6%/55.1%/6.1%/9.2%)	(26.8%/48.0%/9.1%/16.1%)	
HBV-PIS (I/II/III/IV)	15/27/19/37	125/138/66/111	**0.011**
	(15.3%/27.5%/19.4%/37.8%%)	(28.4%/31.4%/15.0%/25.2%)	
AFP, ng/ml, (≤20/>20)	38/60(38.8%/61.2%)	179/261 (40.7%/59.3%)	0.820
Platelet, 10^9^/L, median (range)	141.0 (44.0-408.0)	137.0 (10.0-333.0)	0.157
HBsAg (Positive/Negative)	84/14 (85.7%/14.3%)	363/77 (82.5%/17.5%)	0.551
Tumor number (single/multiple)	90/8 (91.8%/8.2%)	392/48 (89.1%/10.9%)	0.583
Microvascular invasion (yes/no)	22/76 (22.4%/77.6%)	125/315 (28.4%/71.6%)	0.260
Tumor capsule(complete/Inomplete)	54/44 (55.1%/44.9%)	240/200 (54.5%/45.5%)	1.000
Tumor size, cm (≤3/>3)	38/60 (38.8%/61.2%)	199/241 (45.2%/54.8%)	0.262
AJCC stage (I-II/IIIA)	86/12 (87.8%/12.2%)	328/112 (74.5%/25.5%)	**0.005**
BCLC stage (0-A/B-C)	73/25 (74.5%/25.5%)	293/147 (66.6%/33.4%)	0.151

ALT: alanine aminotransferase; AST: aspartate aminotransferase; GGT: gamma-glutamyl transpeptidase; ALB: Albumin; TBIL: total bilirubin; HBV-ALBI: hepatitis B virus load related ALBI score; HBV-PIS: hepatitis B virus load related peritumoral inflammatory score; AFP: alpha fetoprotein; HBsAg: hepatitis B virus surface antigen; AJCC: American Joint Committee on Cancer; BCLC: Barcelona Clinic Liver Cancer.

## Data Availability

The clinical data used to support the findings of this study were provided by Department of Hepatobiliary Surgery, the First and the Second Affiliated Hospital of Chongqing Medical University, and cannot be made freely available. Access to these data will be considered by the author upon request, with permission of directors of Department of Hepatobiliary Surgery of this two hospitals.

## Supplementary Materials

Supplementary Materials
** Figure S1:** K-M survival curves of OS and RFS of PIS and ALBI of 652 patients.** Figure S2: **K-M survival curves of OS and RFS of HBV-PIS and HBV-ALBI in the validation cohorts.** Figure S3:** predictive accuracy comparisons for OS and RFS of 652 patients.** Figure S4:** K-M survival curves of OS and RFS of AJCC and BCLC in the validation cohorts.** Figure S5:** predictive accuracy comparison for OS and RFS in the validation cohorts.** Figure S6: **the calibration curves for OS and RFS in the internal validation cohort.** Figure S7:** the calibration curves for OS and RFS in the external validation cohort.** Figure S8:** predictive accuracy comparison between each variable and the nomograms for OS and RFS in the total of 652 patients.
