# Supplementary material for: HBV-DNA Load-Related Peritumoral Inflammation and ALBI Scores Predict HBV Associated Hepatocellular Carcinoma Prognosis after Curative Resection
Source: J Oncol. 2018 Sep 20;2018:9289421. doi: 10.1155/2018/9289421 (PMC6171205; doi:10.1155/2018/9289421)
Supplement: Supplementary Materials — Figure S1: K-M survival curves of OS and RFS of PIS and ALBI of 652 patients. Figure S2: K-M survival curves of OS and RFS of HBV-PIS and HBV-ALBI in the validation cohorts. Figure S3: predictive accuracy comparisons for OS and RFS of 652 patients. Figure S4: K-M survival curves of OS and RFS of AJCC and BCLC in the validation cohorts. Figure S5: predictive accuracy comparison for OS and RFS in the validation cohorts. Figure S6: the calibration curves for OS and RFS in the internal validation cohort. Figure S7: the calibration curves for OS and RFS in the external validation cohort. Figure S8: predictive accuracy comparison between each variable and the nomograms for OS and RFS in the total of 652 patients. [file 9289421.f1.docx]

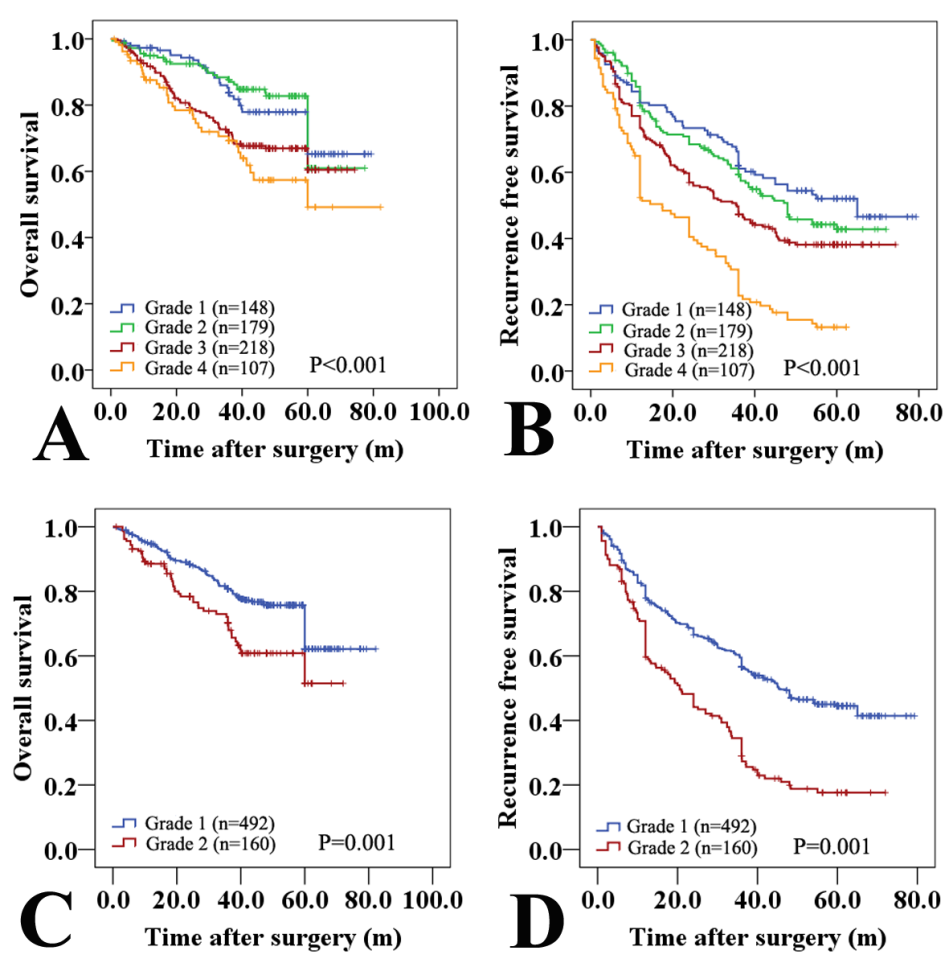


**Figure S1** Kaplan-Meier survival curves of overall survival (OS, A and C) and recurrence-free survival (RFS, B and D) of peritumoral inflammatory score (PIS, A and B) and ALBI (C and D) in the total of 652 patients, respectively.


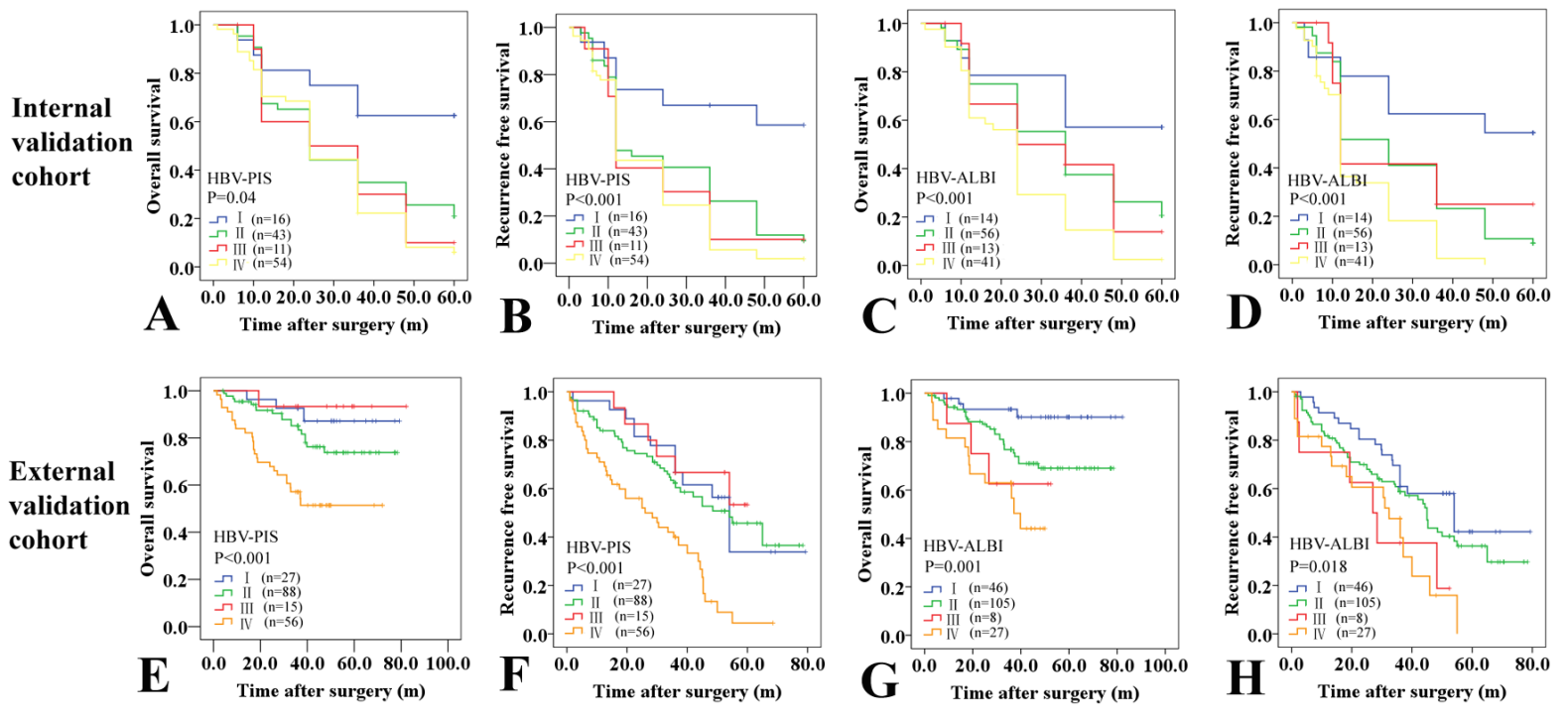


**Figure S2** HBV-DNA load related peritumoral inflammatory score (HBV-PIS) and ALBI score (HBV-ALBI) are associated with overall survival (OS, A, C, E and G) and recurrence-free survival (RFS, B, D, F and H) of patients with hepatocellular carcinoma (HCC) in the internal (A-D) and external validation cohorts (E-H), respectively.


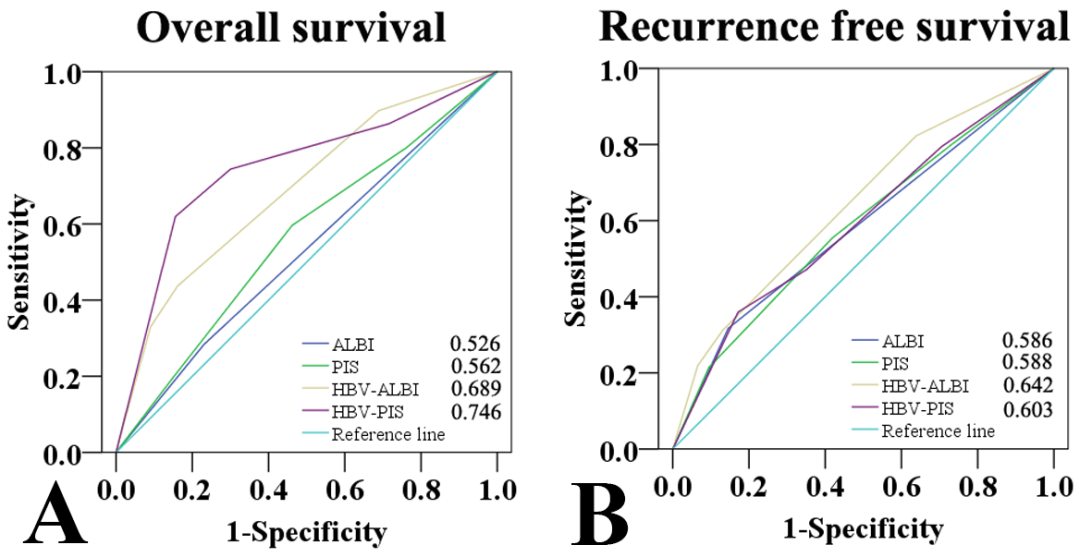


**Figure S3** Predictive accuracy comparisons among peritumoral inflammatory score (PIS), ALBI, HBV-PIS and HBV-ALBI for overall survival (OS, A) and recurrence-free survival (RFS, B) in the total of 652 patients, respectively.


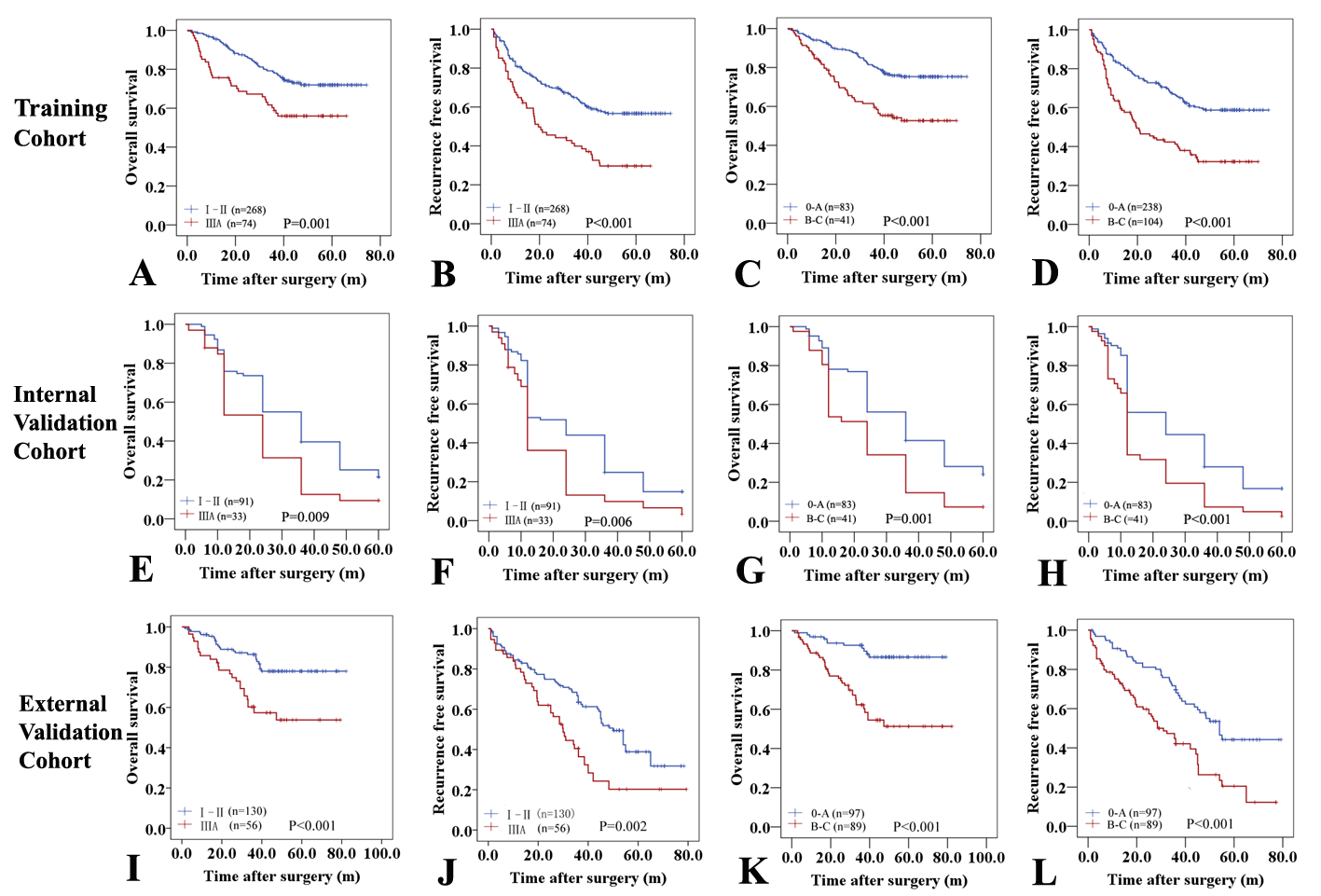


**Figure S4** Kaplan-Meier survival curves of overall survival (OS, A, C, E, G, I and K) and recurrence-free survival (RFS, B, D, F, H, J and L) of AJCC (A, B, E, F, I and J) and BCLC (C, D, G, H, K and L) in the trainning cohort (A-D), the internal (E-H) and external validation cohorts (I-L), respectively.


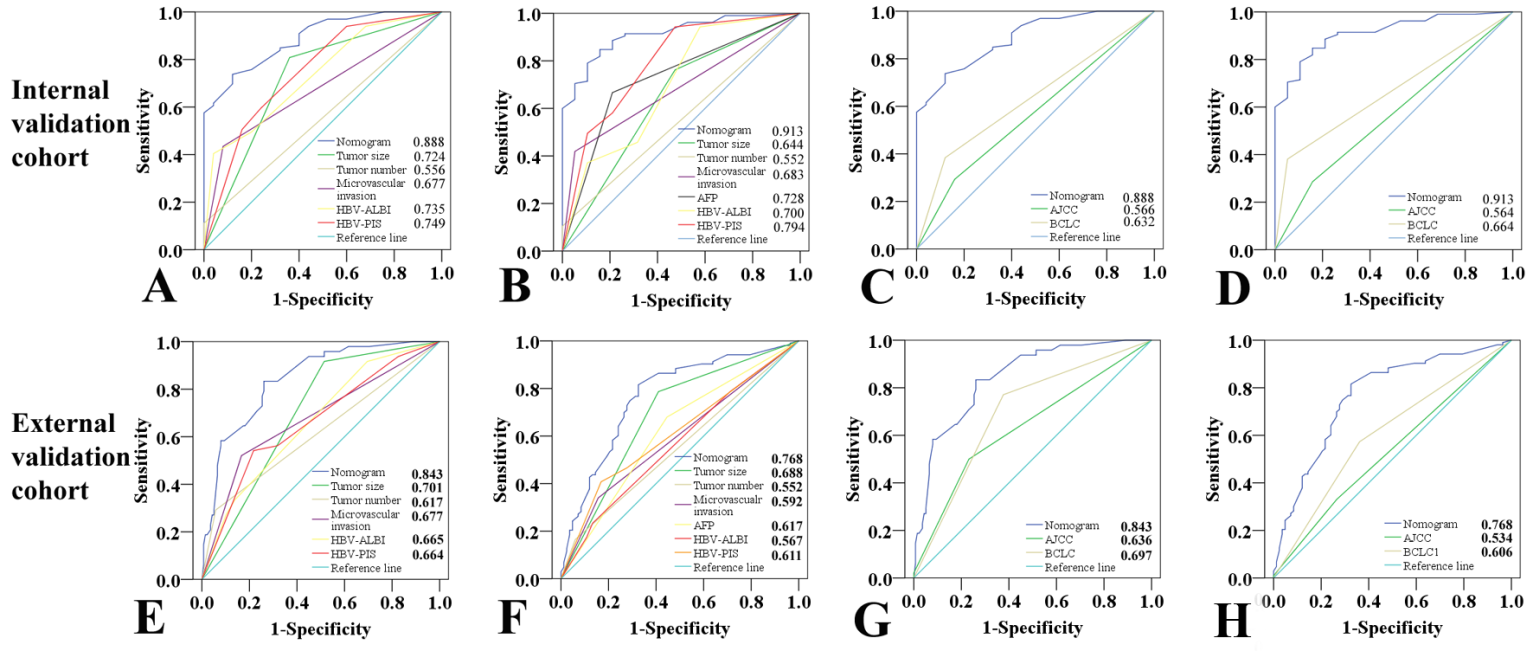


**Figure S5** Predictive accuracy comparison of each variable included in the nomograms (A, B, E and F) and comparison between the nomograms and two conventional clinical staging systems (AJCC and BCLC staging systems, C, D, G and F) by ROC curve analyses for overall survival (OS, A, C, E and G) and recurrence-free survival (RFS, B, D, F and H) in the internal (A-D) and external (E-F) validation cohorts, respectively.


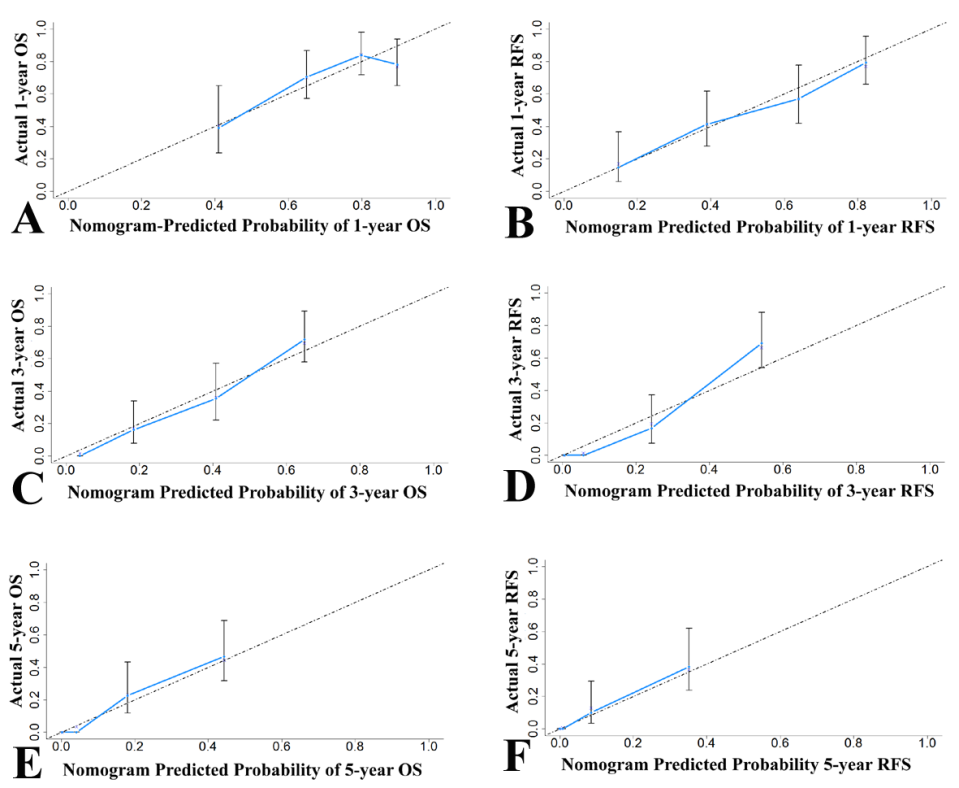


**Figure S6** The calibration curves for predicting 1- (A and B), 3- (C and D) and 5-year (E and F) overall survival (OS, A, C and E) and recurrence-free survival (RFS, B, D and F) rates by nomograms prediction and actual observation in patients with hepatocellular carcinoma in the internal validation cohort, respectively.


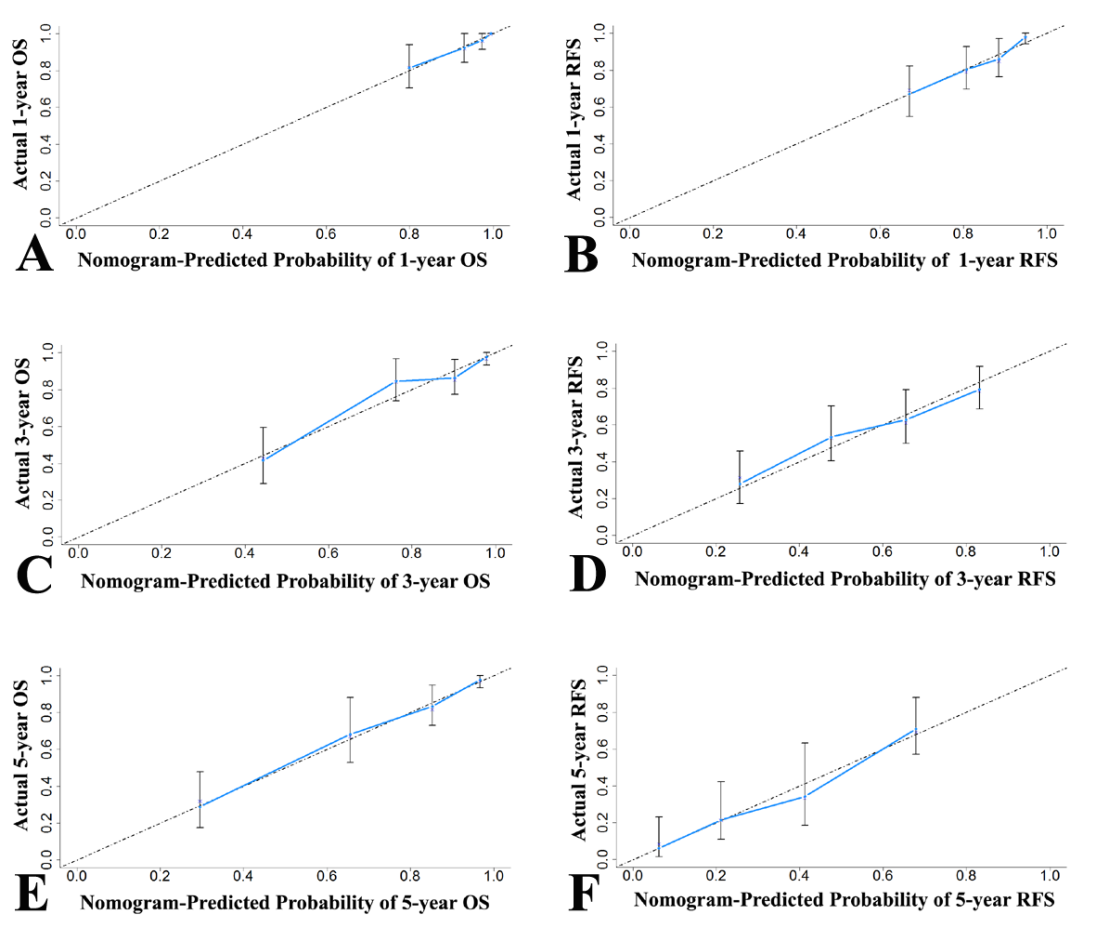


**Figure S7** The calibration curves for predicting 1- (A and B), 3- (C and D) and 5-year (E and F) overall survival (OS, A, C and E) and recurrence-free survival (RFS, B, D and F) rates by nomograms prediction and actual observation in patients with hepatocellular carcinoma in the external validation cohort, respectively.


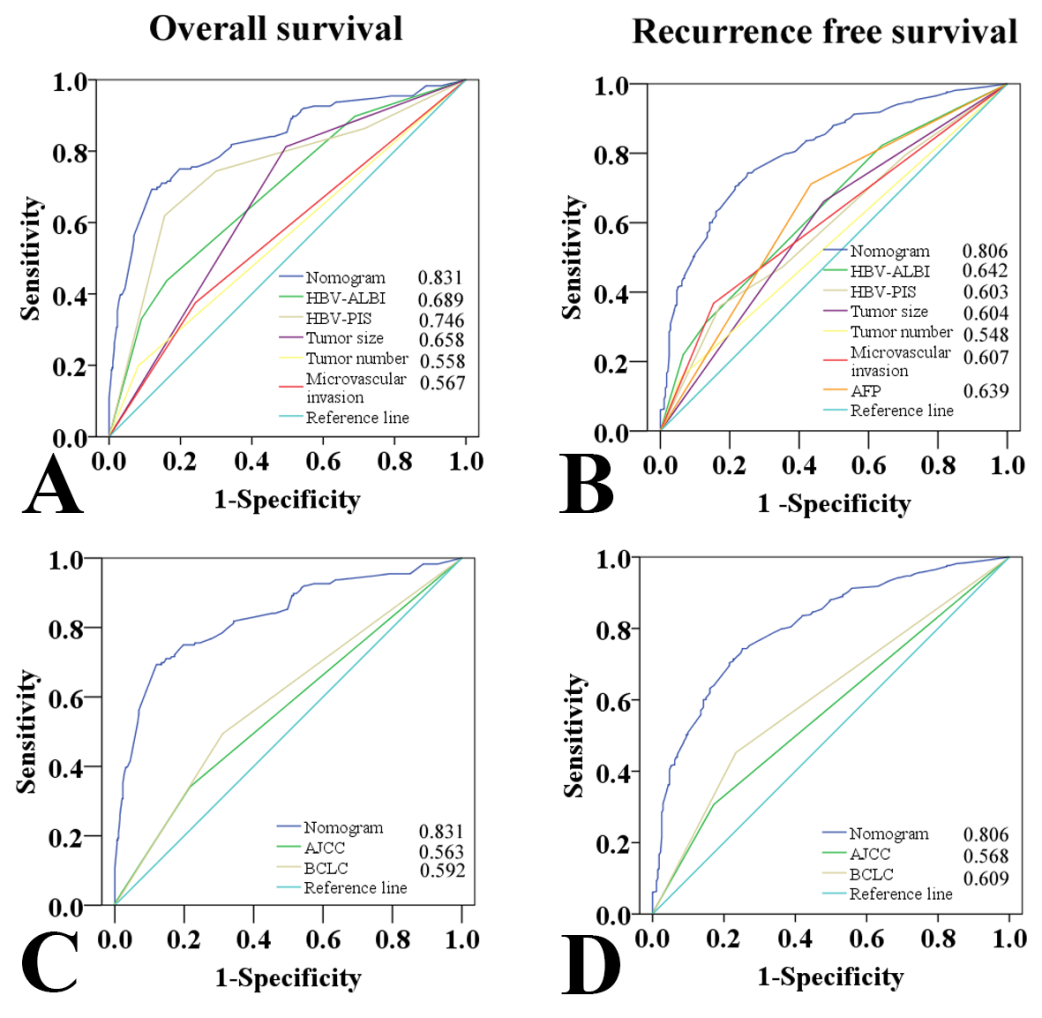


**Figure S8** Predictive accuracy comparison of each variable included in the nomograms (A and B) and comparison between the nomograms and two conventional clinical staging systems (AJCC and BCLC staging systems, C and D) by ROC curve analyses for overall survival (OS, A and C) and recurrence-free survival (RFS, B and D) in the total of 652 patients, respectively.
